# Analysis of Circulating MicroRNAs in Patients with Diabetic Foot Ulcers and Lower Limb Amputation

**DOI:** 10.3390/ijms27083516

**Published:** 2026-04-14

**Authors:** Ricardo Gamboa, Reyna Sámano, Mario Peña-Peña, Vicente Castrejon-Téllez, Alexa Paola Chávez-Nava, Guadalupe Aguilar-Martínez, Claudia Huesca-Gómez

**Affiliations:** 1 Department of Physiology, Instituto Nacional de Cardiología Ignacio Chávez, Mexico City 14380, Mexico; rgamboaa_2000@yahoo.com (R.G.); marionutricion2017@gmail.com (M.P.-P.); vcastrejn@yahoo.com.mx (V.C.-T.); vazquezpao180@gmail.com (A.P.C.-N.); guadalupemaartz@gmail.com (G.A.-M.); 2 Coordinación de Nutrición y Bioprogramación, Instituto Nacional de Perinatología, Mexico City 11000, Mexico; ssmr0119@yahoo.com.mx; 3 Sección de Estudios de Posgrado, Escuela Superior de Medicina, Instituto Politécnico Nacional, Mexico City 11340, Mexico; 4 Unidad Xochimilco, Universidad Autónoma Metropólitana, Mexico City 04960, Mexico

**Keywords:** microRNA expression, diabetes mellitus, diabetic foot ulcers, lower limb amputation

## Abstract

Non-healing diabetic foot ulcers substantially increase the risk of infection and lower limb amputation, contributing to elevated morbidity and mortality among individuals with diabetes. MicroRNAs (miRNAs) have been widely studied as potential early biomarkers for various diseases. This pilot study assessed circulating miRNA expression profiles in adults with diabetic ulcers (*n* = 31), individuals who underwent limb amputation due to foot ulcers (*n* = 20), and 50 control subjects using RT-qPCR. Patients with diabetic foot ulcers and amputations exhibited significantly higher plasma levels of miR-7e, miR-17, miR-33, and miR-191 compared to controls (*p* < 0.05). Notably, miR-191 expression was significantly greater in patients with limb amputations than in those with diabetic ulcers (*p* = 0.001). A strong positive correlation was identified between miR-191 and glucose levels (r = 0.682, *p* = 0.001), while an inverse correlation was observed with HDL-C (r = −0.476, *p* = 0.034). Let-7e also correlated positively with glucose (r = 0.543, *p* = 0.013) and negatively with HDL-C (r = −0.491, *p* = 0.028). Significant associations were found between miR-17-5p and both cholesterol (r = 0.584, *p* = 0.007) and LDL-C (r = 0.495, *p* = 0.026). ROC analyses supported the diagnostic value of these miRNAs: miR-17 and miR-191 achieved AUCs of 0.742 (*p* = 0.000) and 0.671 (*p* = 0.006), respectively, indicating their potential as biomarkers for disease identification. These findings suggest that increased expression of let-7e, miR-17, and miR-191 may serve as significant biomarkers.

## 1. Introduction

Diabetes mellitus (DM) is a metabolic disorder characterized by persistent hyperglycemia resulting from insufficient insulin production or insulin resistance. DM is associated with complications such as retinopathy, nephropathy, neuropathy, peripheral arterial damage, and impaired wound healing [1]. Non-healing diabetic foot ulcers (DFU) substantially increase the risk of infection and lower limb amputation, thereby elevating morbidity and mortality among individuals with diabetes. Diabetic foot disease affects approximately 3–4% of the global population, with the prevalence of foot ulcers among diabetics ranging from 19% to 34% [2]. Approximately 70% of diabetic foot ulcers remain unhealed after 20 weeks of treatment, leading to complications such as ischemia or infection in about 60% of cases. DFUs are a primary cause of amputations [3], although amputation rates vary by country due to genetic and other factors. Diabetes results in long-term complications affecting both microvascular and macrovascular structures. Early detection is critical and the current treatments primarily target blood glucose control. Biomarkers are essential for early diagnosis, prevention, and prediction of disease progression. Advances in molecular biology have facilitated the identification of promising new biomarkers [4,5].

MicroRNAs (miRNAs) are short non-coding RNAs, 20–30 nucleotides long, and are increasingly recognized for their roles in predicting, diagnosing, and treating diseases such as diabetic wound healing [6,7,8]. Although several studies have examined miRNA changes in diabetes [9,10,11], few have focused on the most severe diabetic foot disease (DFU). As a result, research is focusing on the roles of miRNAs in DFU patients. MiRNAs regulate key processes—such as inflammation, angiogenesis, cell migration, and proliferation—that influence wound healing and tissue repair [1].

This study investigated five circulating miRNAs—miR-let-7e-5p, miR-17-5p, miR-33-5p, miR-144-3p, and miR-191-5p—selected based on findings related to DM and DFU from miRNet, PubMed, Scopus, and Google Scholar [7,12,13,14,15]. Few studies have quantified miRNA expression in patients with these conditions. The target genes of these miRNAs are closely associated with glycemic control, diabetic retinopathy, inflammation, and oxidative stress. Accordingly, this pilot study examines circulating miRNA profiles in plasma from adults with diabetic ulcers and those who underwent lower limb amputation, and evaluates these associations. Additionally, metabolic pathways and biological processes were analyzed using in silico methods.

## 2. Results

### 2.1. Characteristics of the Study Population

Table 1 presents the biochemical and anthropometric variables of the study groups. Significant differences were observed among the control group, patients with foot ulcers, and patients with lower limb amputations. Specifically, BMI (*p* = 0.010 and 0.002), total cholesterol (*p* = 0.000 for both), HDL-C (*p* = 0.000 for both), LDL-C (*p* = 0.05 vs. amputation group), glucose (*p* = 0.001 for both), DBP (*p* = 0.005 and 0.001), heart rate (CFx) (*p* = 0.000 for both), and age (*p* = 0.000 and 0.004 when comparing control vs. lower limb amputation and foot ulcers vs. amputation) differed significantly. Table 2 shows the clinical characteristics of amputee patients: 70% were malnourished, 80% had infracondylar amputations. Furthermore, 90% had hypertension.

### 2.2. MicroRNA’s Expression

**Figure 1:**
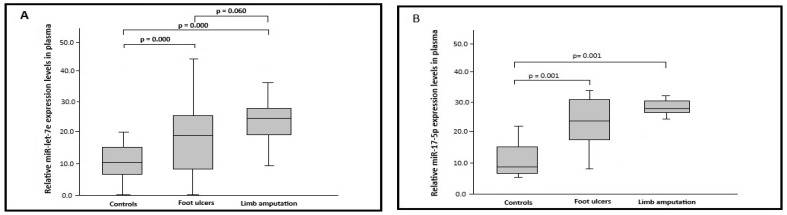

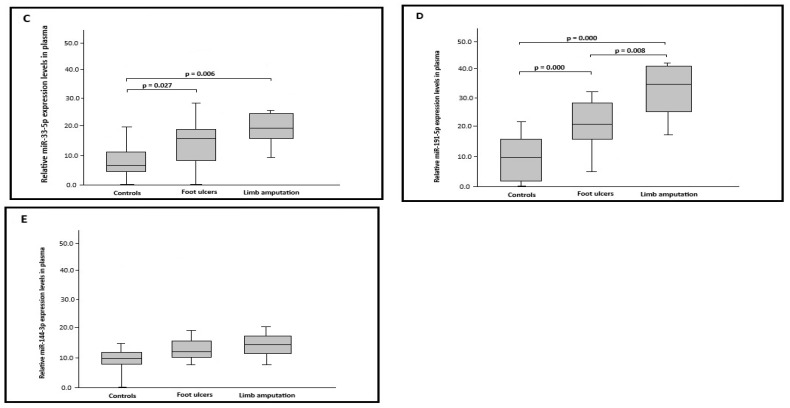
miRNA expression in plasma. Control group, foot ulcer group, and limb amputation group: (**A**) miR-let-7e expression; (**B**) miR-17-5p expression; (**C**) miR-33-5p expression; (**D**) miR-191-5p expression; (**E**) miR-144-3p expression. Data were normalized to cel-miR-39 in plasma and expressed as median with interquartile range.

Plasma miRNAs levels were significantly higher in patients with diabetic ulcers and lower limb amputation than in the control group (Figure 1). Let-7e-5p showed median levels of 10.0 (range 0.51–20.2) in controls; 18.6 (0.28–45.5) in diabetic ulcers; and 24.4 (8.2–35.8) in amputees (*p* = 0.000). miR-17-5p had medians of 8.21 (7.5–23.2) in controls, 23.5 (9.19–33.3) in ulcers, and 29.9 (27.6–31.3) in amputees (*p* = 0.000). miR-191-5p medians were 11.0 (0.43–21.9) in controls, 22.2 (5.6–33.2) in ulcers, and 34.2 (19.1–41.9) in amputees (*p* = 0.000). For miR-33-5p, medians were 8.1 (0.21–19.4) in controls, 18.9 (0.11–29.7) in ulcers, and 21.2 (10.0–24.7) in amputees (*p* = 0.027 and 0.006). miR-144-3p showed 10.3 (0.22–14.3) in controls, 13.5 (9.3–21.1) in ulcers, 15.5 (10.1–22.3) in amputees, with no significant differences (*p* = 1.000). Comparing diabetic ulcers and amputees, only miR-191-5p showed significance (*p* = 0.008). For let-7e, association with amputation approached but did not reach significance (*p* = 0.060).

### 2.3. ROC Curve Analysis

**Figure 2:**
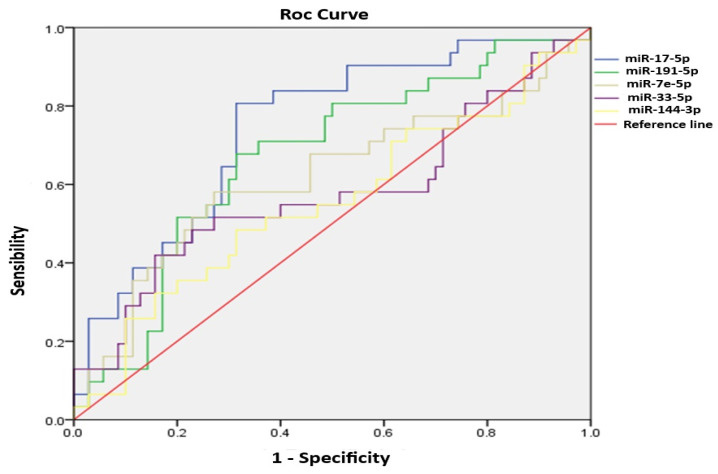
ROC curve analysis. A ROC model was performed to differentiate between the control and patient groups. The whole population was analyzed, 51 diabetic patients and 50 controls.

To evaluate whether the analyzed miRNAs could serve as reliable biomarkers for diagnosing diabetic ulcers or lower limb amputation, ROC curve analyses were performed, and the AUC for each miRNA was determined (see Figure 2). The results showed diagnostic potential for miR-17 (AUC: 0.742 [95% CI, 0.638−0.846, *p*= 0.000]) and miR-191 (AUC: 0.671 [95% CI, 0.556−0.785, *p*= 0.006]), indicating these miRNAs may distinguish affected patients from controls. MiR-7e (AUC: 0.618 [95% CI, 0.488−0.747, *p*= 0.060]), miR-33 (AUC: 0.577 [95% CI, 0.445−0.710, *p*= 0.216]), and miR-144 (AUC: 0.553 [95% CI, 0.425−0.681, *p*= 0.397]) showed lower diagnostic ability.

### 2.4. miRNA Expression Levels and Anthropometric and Biochemical Values

We conducted a correlation analysis of miRNA expression levels with anthropometric and biochemical parameters across the three groups (Table 3). In the control group, a significant sex difference was observed (r = 0.333, *p* = 0.019), along with inverse correlations with systolic blood pressure (r = 0.491, *p* < 0.001) for miR-33 and with HDL-C (r = −0.280, *p* = 0.049) for miR-144. In the diabetic ulcers group, notable inverse correlations were found between triglyceride levels and miR-17, -191, and -144 (r = −0.360, *p* = 0.047; r = −0.361, *p* = 0.046; r = −0.409, *p* = 0.022, respectively). Regarding lower limb amputation, inverse correlations with HDL-C were observed across four miRNAs, with significant results for miR-191 (r = −0.476, *p* = 0.034), miR-7e (r = −0.543, *p* = 0.013), miR-33 (r = −0.681, *p* = 0.001), and miR-144 (r = −0.527, *p* = 0.017). Additionally, positive correlations were identified for all these miRNAs, notably in miR-191 (r = 0.682, *p* = 0.001), miR-7e (r = 0.543, *p* = 0.013), and miR-144 (r = 0.733, *p* < 0.000).

### 2.5. miRNA Expression Levels and Comorbidities

To explore the relationships among miRNA levels, diabetic ulcers, lower limb amputations, and healthy controls, we identified subgroups with the strongest associations with comorbidities (Table 4). miRNA expression values were normalized logarithmically and presented as medians with ranges (min–max) and quartiles 25, 50, 75. We first categorized patients by body mass index into obese and non-obese groups, as well as into categories of hypoalphalipoproteinemia and hypertriglyceridemia. Results indicated that levels of miR-17, miR-7e, and miR-191 were significantly higher in non-obese controls than in patients with foot ulcers. Similarly, these miRNAs were elevated in patients with obesity, hypoalphalipoproteinemia, and hypertriglyceridemia who had foot ulcers or underwent lower limb amputation compared to healthy controls (*p* < 0.005). Additionally, significant differences in miR-191 and miR-7e levels were found between foot ulcer patients and those with amputations, especially in cases of obesity and hypoalphalipoproteinemia, with miR-191 alone higher in patients with hypertriglyceridemia.

### 2.6. Bioinformatic Analysis

**Figure 3:**
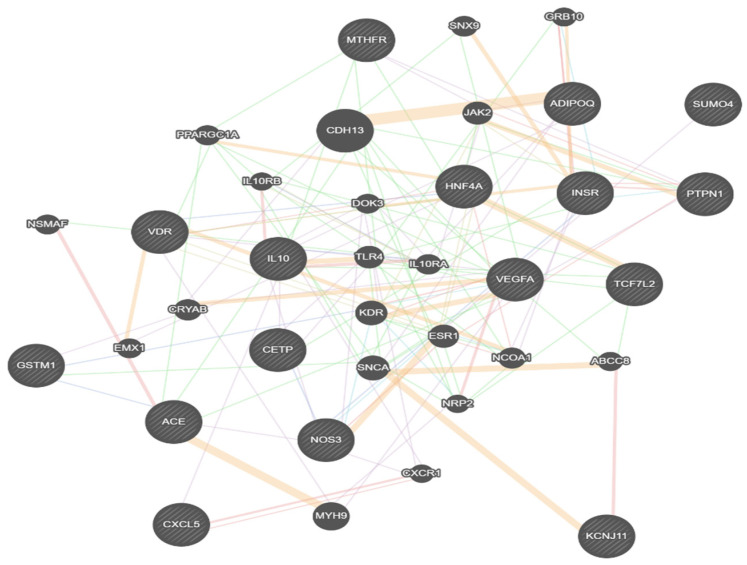
The interaction network generated by GENEmania reveals the role of specific key genes in the development of diabetes and foot ulcers. Each node represents a gene, with lines indicating regulatory interactions.

We also conducted bioinformatic analyses using TargetScan (http://targetscan.org/vert) (accessed on 29 December 2025) to identify genes linked to these miRNAs. The interaction network generated by GeneMANIA then revealed the complexity of molecular interactions, highlighting how their target genes connect within these pathways (see Figure 3).

## 3. Discussion

Diabetic foot ulcers are a major factor in both morbidity and mortality among diabetic patients [1,2]. A key complication is ulceration, which can lead to amputations if not properly managed. miRNAs, small non-coding RNAs, significantly influence the development of diabetes mellitus by affecting insulin secretion, β-cell growth, and function [3]. They are also involved in processes related to diabetic complications, such as angiogenesis, vascularization, inflammation, and signaling pathways. To investigate the relationship between miRNAs in diabetic patients under different conditions, we measured plasma levels of miR-let-7e-5p, miR-17-5p, miR-33-5p, miR-144-3p, and miR-191-5p in patients with foot ulcers, those with lower limb amputations, and healthy controls.

Our analysis showed significant differences in clinical and anthropometric parameters—such as BMI, total cholesterol, HDL-C, diastolic blood pressure, and glucose levels—between control subjects and both patient groups, consistent with previous research. The only significant difference between patients with foot ulcers and those with amputations was age. It is well established that metabolic syndrome (MetS), which includes these factors, is associated with increased risk of diabetes. MetS develops when multiple conditions coexist, raising the future risk of diabetes if left untreated. Although definitions vary, MetS generally involves a combination of cardiometabolic issues—central obesity, high fasting glucose, hypertriglyceridemia, low HDL, and hypertension—which are common in diabetic populations and are reflected in our study groups [16].

Significant differences were observed in the expression of each miRNA studied. Patients with foot ulcers and lower limb amputations exhibit higher levels of let-7e-5p, miR-17-5p, miR-33-5p, and miR-191-5p compared to controls. Additionally, levels of let-7e-5p and miR-191-5p are elevated in patients with foot ulcers compared to those with lower limb amputations. let-7e has shown that genes associated with the insulin signaling pathway are common targets. Several studies have demonstrated that the let-7 family regulates insulin-related gene expression in the pancreas [17], adipocytes [18], and skeletal muscle [19]. Furthermore, it can affect key cellular processes, including proliferation, apoptosis, mitochondrial morphology, cell adhesion, and angiogenesis [20,21,22]. Let-7e has been shown to regulate the expression of the IGF-1 (insulin-like growth factor 1) receptor, which is activated by hormones such as insulin-like growth factor 1 (IGF-1) and IGF-2. It mediates the effects of IGF-1, a polypeptide hormone structurally like insulin. Both IGF-1 and insulin receptors are essential for regulating metabolic and proliferative processes and are involved in endoplasmic reticulum stress, a key factor in tumor growth, insulin resistance, and obesity [23,24]. The overexpression and formation of hybrid receptor isoforms between the IGF-1 receptor and the insulin receptor—sensitive to the three ligands of the IGF axis—as well as hybrid IGF-1 and insulin receptors with other tyrosine kinases, enhance cell transformation, tumor formation, and tumor vascularization. Let-7e-5p targets WNT1 and multiple receptors that initiate signaling cascades in inflammation, including TGFBR1/2 and TLR4. It also targets HMOX1, which reduces oxidative stress and tissue damage [25,26]. miR-17-5p targets TNF-α, a pro-inflammatory cytokine involved in neuroinflammation [27]. Other studies suggested a relationship between miR-17-5p, SMAD7, and TNF-α, in which overexpression of miR-17-5p downregulates SMAD7 expression, increasing TNF-α and other cytokine release [28,29].

Similarly, miR-17-5p is recognized as a strong predictor of metabolic syndrome. Liu et al. [30] found that miR-17-5p improves glucose tolerance and prevents pancreatic β-cell pyroptosis in diabetic mice, likely by targeting the TXNIP/NLRP3 inflammasome pathway. The NLRP3 inflammasome modulates interleukin (IL)-1β and IL-18 production and reduces insulin sensitivity by promoting macrophage-T cell activation in adipose tissue. Additionally, Li et al. [31] reported that exosomes from mesenchymal stem cells can reduce oxidative damage and retinal cell death in diabetic retinopathy mice by transferring miR-17. Evidence has shown that another target of miR-17-5p, HBP1, contributes to premature cellular senescence by either activating p16 or repressing DNMT1, thereby indirectly inhibiting cyclin D1 transcription [32]. Cyclin-D1, encoded by CCND1, is targeted by both Let-7e-5p and miR-17-5p. This protein is overexpressed in senescent cells because it prevents them from entering the S phase [33].

Regarding miR-33-5p, we observed expression, an inverse correlation with HDL-C levels, and an association with obesity and hypoalphalipoproteinemia among subjects who underwent limb amputation. Previous studies have shown that increased expression of miR-33a and miR-144 in monocytes is associated with decreased expression of their target membrane cholesterol transporters, ABCA1 and ABCG1; this may be associated with essential arterial hypertension, regardless of increased carotid intima-media thickness [34]. On the other hand, Xie et al. [35] demonstrated the crucial role of the miR-33-5p/ABCA1/CS axis, along with citrate synthase and cholesterol efflux, in regulating cholesterol flow, inflammation, apoptosis, and aging in vascular endothelial cells (VECs), suggesting this axis could be a target for treating lipid metabolism disorders. These findings may partly explain the link with obesity and hypoalphalipoproteinemia observed in these patients.

While miR-191-5p has been extensively studied in various cancers, where it affects tumor cell apoptosis, proliferation, migration, and cell cycle-related transcription factors [32], it is primarily found in platelets and endothelial cells [36]. Gu Y et al. [37] demonstrated that miR-191 activates NF-kB signaling by increasing p65 mRNA expression. Dangwal et al. [38] found that higher levels of miR-191 promote angiogenesis and enhance the migration of endothelial cells and diabetic dermal fibroblasts. They also observed a positive correlation between miR-191 levels and C-reactive protein and proinflammatory cytokines in patients with type 2 diabetes mellitus [39]. The PPP1CB target gene of hsa-miR-191-5p is associated with the transforming growth factor (TGF)-β signaling pathway, which plays a significant role in aneurysm formation [40,41]. PPP1C interacts with the SMAD2/3 complex and the TGF-β receptor of this pathway, dephosphorylating both and thus controlling their downstream effects. The TGF-β signaling pathway regulates the activation of various genes [40], and its dysregulation or overexpression has been associated with syndromes that cause aneurysms [42]. In our study, we found elevated levels of miR-191-5p, supporting Dangwal’s findings that increased miR-191 can influence angiogenesis and migration in endothelial cells and diabetic dermal fibroblasts. Chronic activation of these processes might exacerbate conditions, potentially leading to diabetic foot ulcers, cell death, and limb amputation. Our higher miR-191-5p expression levels are consistent with Dangwal’s findings, suggesting that elevated miR-191 levels could affect the angiogenic and migratory capacities of these cells. If these processes remain persistent, they may worsen further, resulting in diabetic foot ulcers, cell death, and limb amputation.

Regarding their diagnostic usefulness, ROC curve analysis indicated that miR-17-5p showed strong potential as a biomarker for DFU. This supports other reports that identify miR-17-5p as a classifier for detecting early-risk DFU with high accuracy [20,21]. However, while the biological roles of miR-let-7e and miR-121 are suggested by their overexpression, ROC curve analyses for these miRNAs yielded modest AUC values (0.671 and 0.618, respectively). These moderate figures imply that, although let-7e and miR-121 may serve as initial indicators for DFU, their individual diagnostic power as standalone biomarkers remains limited in our current findings. Therefore, their utility for DFU screening warrants careful assessment and further validation in larger, independent cohorts to confirm their diagnostic capabilities. We examined the relationship between the miRNAs analyzed and the clinical variables. The most notable differences were observed in patients with lower limb amputation for miR-191, let-7e, and miR-144 with HDL-C and glucose levels (*p* < 0.05). Meanwhile, in the second analysis, the relevant parameters were obesity in patients with DFU and miR-191and let-7e.

Previous studies have demonstrated that both miR-191and let-7e expression are positively correlated with blood glucose fluctuations in T2DM [42,43] and are dysregulated in obese individuals with insulin resistance and disrupted glucose homeostasis [44]. Krause et al. [45] found that in patients with metabolic syndrome, let-7e was negatively linked to plasma HDL-C levels and positively associated with features of the syndrome. They also observed significant upregulation of various vascular pathologies. In silico analysis indicated that let-7e plays a key role in regulating the insulin signaling pathway, which is associated with increased insulinemia and elevated HOMA index in our study patients. Furthermore, bioinformatics analysis identified possible complex interactions between altered miRNAs and genes involved in glycemic control, diabetic retinopathy, inflammation, and oxidative stress, providing valuable insights into key pathways and genetic interactions underlying the disease’s pathogenesis.

Limitations and Future Work: To further validate these findings, a larger validation study including more ulcer foot and amputation samples should be conducted to identify additional changes in miRNA expression. This will help assess the sensitivity and specificity of the selected miRNAs as biomarkers for early disease detection. Overall, the miRNAs identified in this pilot study may serve as promising biomarkers for this disease, as there are no prior studies in our population to inform a power analysis for a larger, definitive study. Additional research is needed to explore their role in disease development and their potential as clinical biomarkers.

## 4. Materials and Methods

### 4.1. Patient Population

This pilot study included 101 patients from the Instituto Nacional de Cardiología Ignacio Chávez: 31 with diabetic foot ulcers, 20 with lower limb amputations, and 50 healthy controls. Blood samples were collected from diabetic foot ulcer patients needing debridement or after amputation. Health controls had no symptoms or family history of T2DM, hypertension, or early cardiovascular issues. All participants were Mexican, had at least 3 generations of Mexican ancestry, and were over 40 years old. According to previous studies, the Mexican population is composed of indigenous ancestry (61–66%), European ancestry mainly of Spanish origin (29–32%), and African ancestry (3–5%) [46,47]. All gave informed consent. Participants completed questionnaires on family and medical history, lifestyle, and physical activity. Researchers reviewed their clinical histories as described [48]. Protocol 23-1385 was approved by the Institute’s Research and Ethics Committees. Exclusion criteria were chronic degenerative diseases, cancer, renal disease, familial hypertriglyceridemia, or autoimmune disorders.

### 4.2. Lipid Profile

Enzymatic colorimetric assays measured triglycerides (TG), total cholesterol (TC), and glucose (Roche-Syntex/Boehringer Mannheim, Mannheim, Germany). HDL-C was measured with phosphotungstate/Mg^2+^ (Roche-Syntex) after LDL and VLDL precipitation using the same method. LDL-C was calculated with the Friedewald equation [49], as modified by De Long [50]. All analyses were performed under external quality control (Lipid Standardization Program, CDC, Atlanta, GA, USA). Obesity was defined as a BMI greater than 30 Kg/m^2^. Dyslipidemia was defined according to the National Cholesterol Education Program (NCEP) Adult Treatment Panel III (ATP III) guidelines: TC ≥ 200 mg/dL, LDL-C ≥ 130 mg/dL, HDL-C < 40 mg/dL for men and <50 mg/dL for women, and TG ≥ 150 mg/dL. Dyslipidemia was diagnosed if any of the following criteria were met [51]. Therefore, the parameters of hypoalphalipoproteinemia and hypertriglyceridemia were considered when these values were within the dyslipidemic ranges mentioned above.

### 4.3. Blood and Plasma Sample

Subjects’ blood samples were collected at the time of study enrollment and placed into sterile tubes containing ethylenediaminetetraacetic acid (EDTA). In the case of patients with lower limb amputation, the sample was taken before the surgical amputation procedure. The plasma was immediately centrifuged to separate the plasma for RNA extraction.

### 4.4. RNA Extraction

Total RNA was purified from 200 μL of plasma samples using the miRNeasy Serum/Plasma Kit (Qiagen, Germantown, MD, USA) and further concentrated and purified using the RNA clean-up and Micro-Elute kit (Norgen Biotek, Thorold, ON, Canada) following the manufacturer’s instructions. The A260/280 and A260/230 ratios for the purified RNA were measured with a Nanodrop 8000 spectrophotometer (Thermo Fisher Scientific, Waltham, MA, USA). As an internal control, a synthetic miRNA (cel-miR-39 from *C. elegans*) was added to each sample in equal amounts. Total RNA was stored at −80 °C.

### 4.5. miRNAs Quantitative Real-Time

Reverse transcription was carried out on total plasma RNA using primers for hsa-let-7e-5p, hsa-miR-17-5p, hsa-miR-33-5p, hsa-miR-144-3p, hsa-miR-191-5p, and the TaqMan MicroRNA Reverse Transcription Kit (Thermo Fisher, Waltham, MA, USA) (Primer sequences are detailed in Supplementary Table S1). miRNA levels were quantified with the TaqMan Gene Expression Assay (Thermo Fisher, Waltham, MA, USA) on a CFX96 Touch Real-Time PCR Detection System (Bio-Rad, Hercules, CA, USA). For this reaction, 5 μL of the master mix, 1 μL of PCR primer, and 4 μL of diluted cDNA were used. PCR cycling conditions included 2 min at 50 °C, 10 min at 95 °C, followed by 40 cycles of 15 s at 95 °C and 60 s at 60 °C. The amplification curves were analyzed using ABI SDS software, V2.2 to determine Ct values. The gene expression levels of the selected miRNAs are shown as ΔCt relative to the mean Ct values of external references, including cel-miR-39. Fold change was calculated relative to the group of healthy individuals. All groups used this normalization method. Relative expression levels were computed using the 2-ΔΔCT method [52].

### 4.6. Statistical Analysis

Data analysis was performed using SPSS v21 (https://www.ibm.com/mx-es/products/spss-statistics) (accessed on 9 December 2025). For RT-PCR data analysis, normalized Ct values (ΔCt) were calculated for each sample using the mean threshold of the miRNA of interest and the endogenous control. The Kolmogorov–Smirnov test was used to evaluate the normality of each variable. Quantitative variables are presented as mean ± standard deviation (S.D.), while qualitative variables are expressed as frequencies and percentages. We used Student’s *t*-test or the U-Mann–Whitney test to compare two groups. The Kruskal–Wallis test was used for comparing more than two groups. The diagnostic performance of miRNA expression was assessed using ROC curves and AUC calculations. Youden’s index was used to determine the optimal cutoff point for each miRNA. Results are reported as odds ratios (OR) with 95% confidence intervals. A *p*-value less than 0.05 was considered statistically significant.

### 4.7. Bioinformatic Analysis

To predict potential target genes for miRNAs hsa-let-7e-5p, hsa-miR-17-5p, hsa-miR-33-5p, hsa-miR-144-3p, and hsa-miR-191-5p, we employed multiple computational algorithms. These algorithms identify target genes by matching the seed region of a miRNA (nucleotides 2–7) with the mRNA’s 3′ untranslated region (3′ UTR). The databases used for this analysis were: miRNet (http://www.mirnet.ca, v2.0), mirdb.org/miRDB, TargetScan v7.2 (http://www.targetscan.org/vert_80/), and GeneMANIA (https://genemania.org) accessed on 29 Decembre 2025.

## 5. Conclusions

In conclusion, our results revealed differences in the expression of the studied miRNAs. Identifying the mechanisms behind complications at both the molecular and cellular levels is essential for a better understanding of why some people with diabetes develop more severe diabetic foot issues and what factors lead to limb amputation, even when receiving the same treatment and care. The detailed molecular mechanisms by which different genes in the regulatory network contribute to the development of diabetes and its complications remain unclear and require further investigation. These findings are significant, as few studies have explored the relationship between miRNA expression levels in patients with foot ulcers and limb amputations.

## Figures and Tables

**Table 1:** Clinical characteristics in the three studied groups.

Variable	Controls *n* = 50	Foot Ulcers *n*= 31	Lower Limb Amputation *n* = 20	P1	P2	P3
Age (years)	47.8 ± 5.1	53.2 ± 13.3	62.5 ± 11.7	0.052	0.000	0.004
Sex (M/F)	30/20			0.573	0.071	0.911
Weight (Kg)	69.9 ± 11.5	73.8 ± 16.7	78.8 ± 14.5	0.694	0.051	0.670
BMI (Kg/m^2^)	25.1 ± 2.9	28.3 ± 5.7	29.3 ± 6.0	0.010	0.002	1.000
TC (mg/dL)	186.22 ± 37.5	223.6 ± 47.9	243.9 ± 33.0	0.000	0.000	0.257
HDL-C (mg/dL)	46.4 ± 12.6	34.4 ± 2.9	35.2 ± 3.1	0.000	0.000	1.000
LDL-C (mg/dL)	113.47 ± 32.2	130.4 ± 18.3	139.7 ± 39.4	0.139	0.025	1.000
Tryglicerides (mg/dL)	137.2 ± 77.9	136.4 ± 18.3	134.7 ± 19.3	1.000	1.000	1.000
Glucose (mg/dL)	92.6 ± 8.8	129.2 ± 16.2	152.0 ± 61.5	0.001	0.000	0.180
SBP (mmHg)	127.3 ± 20.2	124.2 ± 16.2	125.0 ± 12.7	1.000	1.000	1.000
DBP (mmHg)	86.4 ± 20.4	79.9 ± 9.0	73.3 ± 11.4	0.005	0.013	1.000
CFx (bm)	77.0 ± 12.1	101.7 ± 32.2	115.2 ± 21.0	0.000	0.000	0.102

BMI: Body Mass Index; TC: Total Cholesterol; HDL-C: High-Density Lipoprotein Cholesterol; LDL-C: Low-Density Lipoprotein Cholesterol; SBP: Systolic Blood Pressure; DBP: Diastolic Blood Pressure; CFx: Cardiac Frequency. P1 = Control vs. Foot ulcers, P2 = Control vs. Lower limb amputation, P3 = Foot ulcers vs. Lower limb amputation.

**Table 2:** Clinical characteristics of the group with lower limb amputation.

Amputation Group	
Sex Women/Men %	30/70
Supracondylar/Infracondylar %	20/80
Evolution Years medium (Supracondylar/Infracondylar)	9.5/6.3
Wagner Grade	Grade V
(SAH) %	90
Smoking %	20
Medication	Insulin, Metformin, Irbesartan, Hydrochlorothiazide, atorvastatin, ACEI, beta blockers

SAH: systemic arterial hypertension, ACEI: angiotensin converting enzyme inhibitors.

**Table 3:** Pearson correlation between clinical characteristics and miRNAs.

Controls										
	Age	Sex	BMI	TC	HDL-C	LDL-C	TG	Glucose	SBP	DBP
miR-17	0.080 (0.580)	0.145 (0.316)	−0.047 (0.744)	0.050 (0.729)	0.001 (0.995)	−0.014 (0.923)	0.113 (0.436)	−0.086 (0.554)	0.012 (0.934)	−0.052 (0.718)
miR-19	−0.105 (0.469)	0.055 (0.705)	−0.101 (0.487)	−0.098 (0.496)	0.107 (0.460)	−0.267 (0.150)	0.044 (0.763)	−0.163 (0.269)	0.086 (0.553)	0.054 (0.709)
miR-7e	−0.008 (0.957)	−0.027 (0.853)	−0.127 (0.381)	−0.215 (0.134)	0.058 (0.687)	0.243 (0.089)	−0.129 (0.371)	−0.203 (0.157)	0.019 (0.897)	−0.002 (0.989)
miR-33	0.256 (0.673)	0.333 (0.019)	0.034 (0.814)	0.171 (0.235)	0.083 (0.567)	0.163 (0.259)	0.044 (0.764)	−0.033 (0.822)	−0.491 (0.000)	−0.115 (0.430)
miR-144	0.128 (0.374)	−0.009 (0.374)	0.253 (0.076)	−0.096 (0.509)	−0.280 (0.049)	−0.059 (0.049)	0.128 (0.377)	0.021 (0.885)	−0.192 (0.182)	0.235 (0.105)
Foot ulcers										
	Age	Sex	BMI	TC	HDL-C	LDL-C	TG	Glucose	SBP	DBP
miR-17	−0.064 (0.745)	−0.094 (0.629)	−0.083 (0.679)	0.138 (0.457)	0.076 (0.683)	0.149 (0.423)	−0.360 (0.047)	−0.094 (0.616)	−0.073 (0.713)	0.049 (0.803)
miR-191	−0.034 (0.864)	0.223 (0.346)	0.218 (0.275)	0.088 (0.637)	0.0105 (0.575)	−0.091 (0.624)	−0.361 (0.046)	−0.280 (0.128)	−0047 (0.698)	−0.010 (0.961)
miR-7e	−0.004 (0.485)	−0.266 (0.163)	−0.308 (0.118)	0.110 (0.566)	0.078 (0.676)	−0.044 (0.812)	0.342 (0.060)	−0.181 (0.331)	−0.125 (0.528)	−0.081 (0.683)
miR-33	0.308 (0.110)	−0.284 (0.135)	−0.201 (0.316)	−0.196 (0.292)	0.224 (0.228)	−0.345 (0.057)	−0.034 (0.855)	0.041 (0.826)	−0.143 (0.469)	−0.377 (0.048)
miR-144	−0.013 (0.375)	−0.068 (0.727)	−0.191 (0.340)	0.123 (0.510)	0.069 (0.711)	0.105 (0.573)	−0.409 (0.022)	−0.136 (0.466)	−0.024 (0.905)	−0.001 (0.998)
Lower limb amputation										
	Age	Sex	BMI	TC	HDL-C	LDL-C	TG	Glucose	SBP	DBP
miR-17	−0.087 (0.716)	−0.006 (0.981)	0.311 (0.182)	0.584 (0.007)	−0.129 (0.588)	0.495 (0.026)	−0.315 (0.176)	0.123 (0.604)	0.337 (0.171)	0.163 (0.517)
miR-191	0.236 (0.333)	0.317 (0.173)	−0.195 (0.410)	0.183 (0.440)	−0.476 (0.034)	0.052 (0.827)	−0.192 (0.418)	0.682 (0.001)	−0.155 (0.539)	0.327 (0.186)
miR-7e	0.231 (0.327)	0.280 (0.232)	−0.168 (0.480)	0.239 (0.309)	−0.491 (0.028)	−0.053 (0.824)	−0.100 (0.676)	0.543 (0.013)	−0.097 (0.703)	−0.356 (0.147)
miR-33	0.085 (0.721)	0.309 (0.185)	0.039 (0.870)	0.094 (0.694)	−0.681 (0.001)	−0.380 (0.098)	0.062 (0.796)	0.372 (0.107)	−0.194 (0.441)	−0.212 (0.399)
miR-144	0.158 (0.505)	0.397 (0.083)	−0.333 (0.156)	−0.133 (0.635)	−0.527 (0.017)	−0.344 (0.134)	−0.143 (0.547)	0.733 (0.000)	−0.085 (0.737	−0.262 (0.294)

BMI: Body mass index TC: total cholesterol, HDL-C: High density lipoprotein cholesterol, LDL-C: Low density lipoprotein cholesterol, TG: Triglycerides, SBP: Systolic blood pressure, DBP: Diastolic blood pressure.

**Table 4:** miRNA expression values are normalized using logarithms.

Without Obesity	Controls Median (Min–Max) Q1, Q2, Q3	Foot Ulcers Median (Min–Max) Q1, Q2, Q3	Lower Limb Amputation Median (Min–Max) Q1, Q2, Q3	P1	P2	P3
miR-17	1.07 (0.36–2.32) 0.48–1.07–1.95	2.32 (1.19–3.38) 1.51–2.32–2.88	2.24 (1.36–2.95) 1.65–2.24–2.70	0.002	0.053	1.000
miR-191	1.07 (0.74–1.35) 0.55–1.07–1.23	2.79 (1.24–4.33) 1.88–2.79–3.63	1.85 (1.02–2.35) 1.66–1.85–2.05	0.002	0.024	1.000
miR-7e	1.13 (0.92–1.34) 0.96–1.13–1.23	2.32 (0.92–3.72) 1.25–2.32–3.12	2.16 (0.51–3.82) 1.21–2.16–3.33	0.016	0.205	1.000
miR-33	0.99 (0.82–1.17) 0.85–0.99–1.05	1.60 (0.98–2.21) 1.23–1.60–1.88	1.43 (0.44–3.31) 0.78–1.43–2.78	0.056	0.615	1.000
miR-144	1.05 (0.93–1.18) 0.97–1.05–1.10	1.21 (0.16–2.25) 0.45–1.21–1.98	1.22 (0.53–2.99) 0.88–1.22–2.32	1.000	1.000	1.000
With obesity	Controls Median (min–max) Q1, Q2, Q3	Foot ulcers Median (min–max) Q1, Q2, Q3	Lower limb amputation Median (min–max) Q1, Q2, Q3	P1	P2	P3
miR-17	0.93 (0.72–1.13) 0.84–0.93–1.09	2.30 (1.63–2.48) 1.88–2.30–2.39	2.77 (2.32–3.01) 2.46–2.77–2.91	0.000	0.000	0.086
miR-191	0.92 (0.67–1.28) 0.84–0.92–1.21	2.05 (1.63–2.48) 1.82–2.05–2.26	3.14 (2.67–3.60) 2.84–3.14–3.22	0.000	0.000	0.000
miR-7e	0.87 (0.65–1.09) 0.76–0.87–1.01	1.62 (1.17–2.06) 1.35–1.62–1.88	2.43 (2.04–2.83) 2.23–2.43–2.65	0.004	0.000	0.005
miR-33	1.00 (0.78–1.21) 0.86–1.00–1.14	1.44 (0.83–2.05) 1.09–1.44–1.74	1.67 (1.33–2.01) 1.48–1.68–1.88	0.252	0.039	1.000
miR-144	0.94 (0.75–1.13) 0.83–0.94–1.06	1.06 (0.79–1.32) 0.90–1.06–1.19	1.14 (0.73–1.56) 0.88–1.14–1.32	1.000	0.825	1.000
Hipo-α	Controls Median (min–max) Q1, Q2, Q3	Foot ulcers Median (min–max) Q1, Q2, Q3	Lower limb amputation Median (min–max) Q1, Q2, Q3	P1	P2	P3
miR-17	0.91 (0.38–1.62) 0.54–0.91–1.48	2.25 (1.26–3.38) 1.62–2.25–3.02	2.66 (1.36–3.33) 1.99–2.66–3.09	0.000	0.000	0.063
miR-191	0.83 (0.43–1.75) 0.63–0.83–1.56	2.25 (1.26–5.32) 1.65–2.25–4.78	3.08 (1.31–4.49) 2.37–3.08–4.10	0.000	0.000	0.010
miR-7e	0.90 (0.51–1.58) 0.65–0.90–1.39	1.80 (1.58–4.55) 1.65–1.80–3.69	2.38 (0.82–3.58) 1.34–2.38–3.21	0.009	0.000	0.028
miR-33	1.01 (0.12–1.71) 0.39–1.01–1.56	1.45 (1.10–5.47) 1.30–1.45–4.86	1.62 (0.02–2.47) 0.88–1.62–2.13	0.058	0.006	0.511
miR-144	1.14 (0.22–2.10) 0.44–1.14–1.89	1.10 (0.20–2.65) 0.57–1.10–2.24	1.16 (0.13–2.51) 0.52–1.16–2.22	0.884	0.908	0.806
HiperTG	Controls Median (min–max) Q1, Q2, Q3	Foot ulcers Median (min–max) Q1, Q2, Q3	Lower limb amputation Median (min–max) Q1, Q2, Q3	P1	P2	P3
miR-17	1.03 (0.35–2.32) 1.51–1.03–2.06	2.02 (1.19–3.19) 1.65–2.02–2.88	2.34 (1.36–3.02) 1.69–2.35–2.78	0.000	0.000	0.206
miR-191	1.05 (0.43–2.19) 0.63–1.05–1.88	1.77 (1.26–3.01) 1.39–1.77–2.59	2.70 (1.31–4.49) 1.88–2.70–3.99	0.000	0.000	0.028
miR-7e	0.84 (0.51–2.02) 0.60–0.84–1.89	1.39 (0.58–2.66) 0.99–1.39–2.11	1.98 (0.82–3.58) 1.22–1.98–2.87	0.014	0.000	0.318
miR-33	1.00 (0.12–1.71) 0.41–1.00–1.58	1.71 (0.46–2.70) 0.58–1.71–2.33	1.48 (0.96–1.83) 1.21–1.48–1.65	0.308	0.120	1.000
miR-144	1.09 (0.36–1.60) 0.56–1.09–1.38	0.68 (0.20–1.79) 0.45–0.68–1.23	0.89 (0.13–2.51) 0.48–0.89–2.11	1.000	1.000	1.000

The values are presented as median (min–max). and the quartiles are 25, 50, and 75. P1: Controls vs. Foot ulcers; P2: Controls vs. Lower limb amputation; P3: Foot ulcers vs. Lower limb amputation. Hipo-α: Hypoalphalipoproteinemia, HyperTG: Hypertriglyceridemia.

## Data Availability

The data presented in this study are not publicly available due to ethical restrictions. They are only available on request from the corresponding author.

## Supplementary Materials

The following supporting information can be downloaded at: https://www.mdpi.com/article/10.3390/ijms27083516/s1.
