# Supplementary material for: Analysis of Circulating MicroRNAs in Patients with Diabetic Foot Ulcers and Lower Limb Amputation
Source: Int J Mol Sci. 2026 Apr 14;27(8):3516. doi: 10.3390/ijms27083516 (PMC13116869; doi:10.3390/ijms27083516)
Supplement: Supplementary file 1 [file ijms-27-03516-s001.zip › ijms-4213837-supplementary.pdf]

miRNA	*Primer sequence*	*Annealing temperature (qPCR)*
let-7e-5p	*TGAGGTAGGAGGTTGTATAGTT*	*60°C*
miR-17-5p	*CAAAGTGCTTACAGTGCAGGTAG*	*60°C*
miR-33-5p	*GTGCATTGTAGTTGCATTGCA*	*60°C*
miR-144-3p	*TACAGTATAGATGATGTACT*	*60°C*
miR-191-5p	*AACGGAATCCCAAAAGCAG*	*60°C*

Table S1. Sequence of primers used.
